# Long-term survival with eribulin in an elderly patient with inoperable retroperitoneal well-differentiated liposarcoma: a case report and literature review

**DOI:** 10.3389/fonc.2026.1777562

**Published:** 2026-04-21

**Authors:** Jiwen Fan, Meijing Chen, Qunyao Dai, Boran Cheng, Wenjuan Lai, Shubin Wang, Gangling Tong

**Affiliations:** 1 Department of Oncology, Peking University Shenzhen Hospital, Affiliated to Shenzhen University, Shenzhen Key Laboratory of Gastrointestinal Cancer Translational Research, Cancer Institute of Shenzhen-Peking University-Hong Kong University of Science and Technology (PKU-HKUST) Medical Center, Shenzhen, Guangdong, China; 2 Department of Radiology, Peking University Shenzhen Hospital, Shenzhen, Guangdong, China; 3 Nursing Department, Peking University Shenzhen Hospital, Shenzhen, Guangdong, China

**Keywords:** elderly, erbulin, liposarcoma, retroperitoneal neoplasms, well-differentiated liposarcoma

## Abstract

**Background:**

The management of retroperitoneal well-differentiated liposarcoma (WDLPS) in elderly, surgically ineligible patients remains a therapeutic challenge. Eribulin, a microtubule inhibitor, has demonstrated activity in advanced non-WDLPS liposarcoma subtypes; however, its efficacy in pure well-differentiated liposarcoma (WDLPS) remains exploratory, and long-term outcomes in elderly patients are not well documented.

**Case presentation:**

An 82-year-old female presented with a large retroperitoneal mass. Biopsy confirmed WDLPS with MDM2 amplification. Due to age, comorbidities, and unresectable disease, first-line therapy with anlotinib was initiated. However, treatment was complicated by a hypertensive crisis and radiological evidence of slow disease progression. Therapy was switched to second-line eribulin (1.4 mg/m² on days 1 and 8 of a 21-day cycle).

**Results:**

The patient has maintained sustained disease stability for over five years while receiving continuous eribulin treatment, as confirmed by serial CT imaging assessed using RECIST criteria version 1.1. Treatment has been well tolerated, with adverse events limited to Grade I–II leukopenia and no significant neurotoxicity or gastrointestinal effects. The patient’s quality of life and glucose homeostasis (Doege-Potter syndrome-associated hypoglycemia) have been well preserved throughout this period.

**Conclusion:**

This case suggests that eribulin may provide durable disease control and is a well-tolerated therapeutic option for elderly patients with inoperable retroperitoneal WDLPS, providing preliminary evidence for its potential to achieve long-term tumor stability and maintain quality of life in this vulnerable population. Multidisciplinary assessment and individualized treatment strategies remain essential in optimizing outcomes.

## Introduction

Retroperitoneal liposarcoma (RLPS) is a rare malignant neoplasm originating from the retroperitoneal space, accounting for 0.07%–0.2% of all malignant tumors and 12%–40% of all liposarcomas, representing the predominant pathological subtype among retroperitoneal sarcomas (1). The clinical onset of RLPS is insidious, often lacking obvious symptoms in its early stages. By the time patients present with noticeable abdominal masses, distension, pain, or weight loss, tumors are typically already large or locally advanced (2). Furthermore, the average tumor diameter at diagnosis usually exceeds 15 cm, and some cases may even surpass 30 cm, posing significant therapeutic challenges (3). According to the World Health Organization (WHO) classification, RLPS is histologically divided into four subtypes: well-differentiated liposarcoma (WDLPS), dedifferentiated liposarcoma (DDLPS), myxoid liposarcoma (MLPS), and pleomorphic liposarcoma (PLPS). Among these, WDLPS and DDLPS are the most common, comprising 37%–56% of primary retroperitoneal liposarcomas (4). Both subtypes are characterized by amplification of the MDM2 (murine double minute 2) gene on chromosome 12q13-15, which serves as a highly sensitive and specific diagnostic marker (5). However, despite this shared molecular feature, WDLPS and DDLPS exhibit divergent clinical trajectories: WDLPS typically follows an indolent course with low metastatic potential but high local recurrence risk, while DDLPS is associated with more aggressive behavior, higher metastatic potential, and poorer prognosis (6). This distinction is critical when evaluating responses to systemic therapy, as clinical outcomes with agents such as eribulin can vary significantly between these subtypes. Currently, surgical resection remains the most effective treatment for RLPS, with complete (R0) resection being a key determinant of patient outcomes. Studies have indicated that following conventional surgical resection, the 3-year recurrence-free survival (RFS) and overall survival (OS) rates are 32.4% and 83.8%, respectively (7). However, due to the complex retroperitoneal anatomy and frequent proximity of tumors to vital vessels and organs, surgical difficulty is often high, R0 resection rates remain low, and postoperative local recurrence rates can reach 40%–80%. Treatment options are particularly limited for patients with unresectable disease or those of advanced age (8).

For unresectable or advanced RLPS, systemic therapy becomes the mainstay of management, including conventional chemotherapy, multi−target kinase inhibitors, and novel microtubule inhibitors. Anlotinib, a multi−target tyrosine kinase inhibitor, effectively suppresses targets such as VEGFR and FGFR (9). In a study of advanced soft−tissue sarcomas, anlotinib as first−line therapy yielded median progression−free survival (PFS) and OS of 8.71 months and 16.23 months, respectively, in the liposarcoma subgroup (10). Eribulin, a microtubule dynamics inhibitor, has shown subtype-specific activity in advanced liposarcoma, primarily in non-WDLPS subtypes. In a pivotal phase III trial comparing eribulin with dacarbazine, subgroup analysis of patients with advanced dedifferentiated, myxoid/round cell, or pleomorphic liposarcoma demonstrated significant improvements in both OS (18.7 vs. 6.2 months, P < 0.001) and PFS (2.9 vs. 1.7 months, P = 0.0015) with a comparable safety profile (11). It is important to note, however, that patients with pure WDLPS were excluded from this trial, therefore, the observed efficacy cannot be directly extrapolated to this subtype. Subsequent translational studies have explored potential mechanisms underlying this differential sensitivity, including alterations in microtubule dynamics and tumor microenvironment in both WDLPS and DDLPS (12). Despite these advances, reports on the long-term use of eribulin in elderly patients with well-differentiated RLPS remain scarce, highlighting the need for further clinical evidence in this specific population.

This paper reports the case of an 82−year−old female with a large, well−differentiated retroperitoneal liposarcoma harboring MDM2 amplification, who maintained tumor stability for over 5 years during continuous eribulin treatment. This experience suggests that eribulin may offer favorable efficacy and tolerability in elderly patients with well−differentiated RLPS and provides new clinical evidence for managing such refractory cases.

## Case presentation

An 82-year-old female patient with a documented history of hypertension and non-contributory family history presented to the hospital in April 2020 with unexplained dizziness and unsteady gait. No relevant psychosocial factors or genetic predisposition were identified. Physical examination revealed significant abdominal distension, with a diffuse, firm, ill-defined, non-tender mass palpable throughout the abdomen. There was no rebound tenderness, bowel sounds were normal, and the fluid wave test was negative. No prior interventions had been performed for the retroperitoneal mass before this presentation.

**Figure 1:**
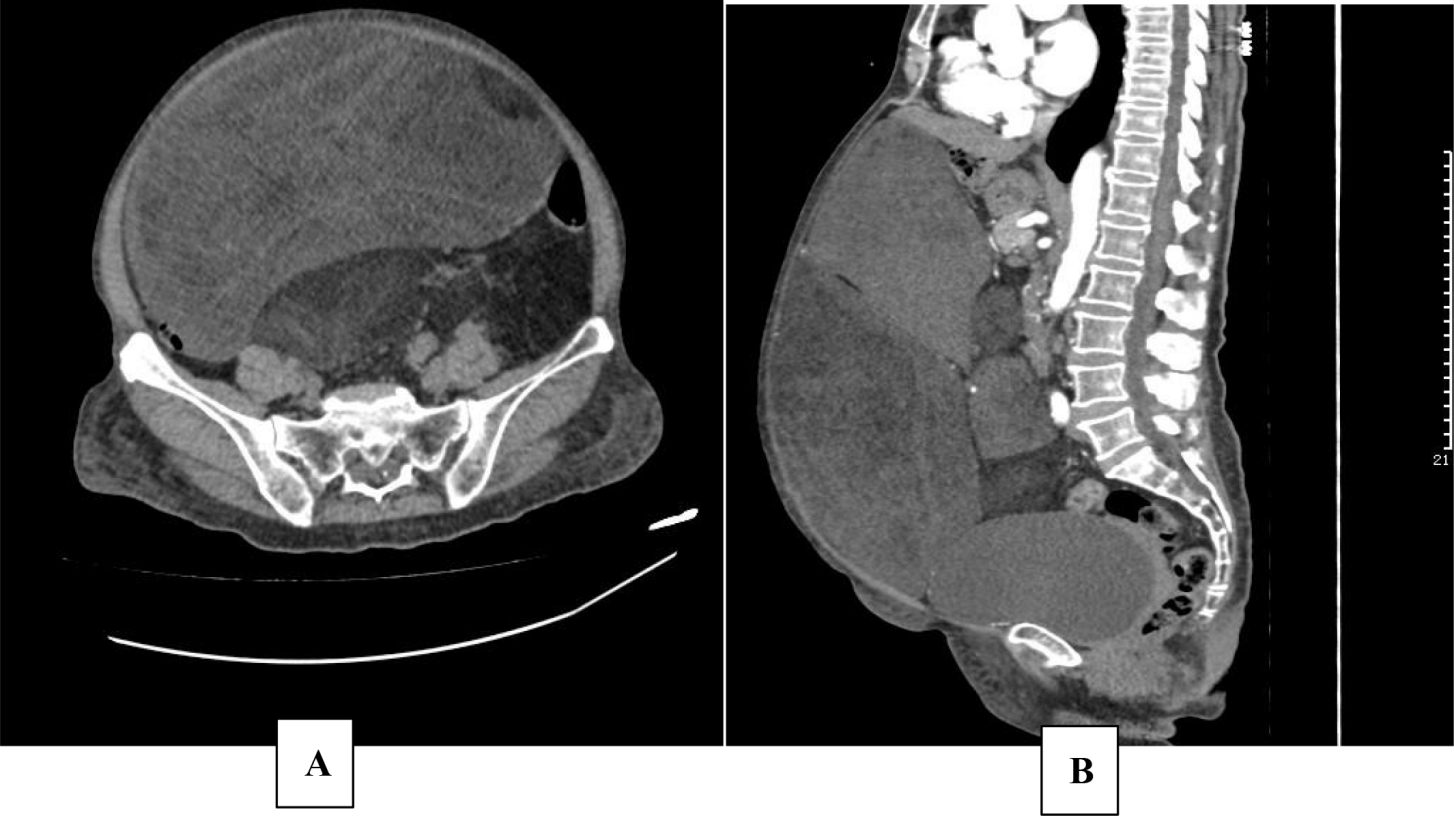
Abdominal CT. Abdominal CT obtained in April 2020 (before therapy). Axial **(A)**; Sagittal **(B)**.

Laboratory investigations at admission indicated significant hypoglycemia, with blood glucose measured at 1.87 mmol/L. Complete blood count (CBC), liver and kidney function tests, electrolyte levels, and cardiac enzyme profiles were all within normal ranges. Brain magnetic resonance imaging (MRI) revealed no space-occupying lesions. Echocardiography indicated essentially normal cardiac structure and function. Abdominal computed tomography (CT) scan identified multiple low-density lesions in the abdomen. Due to the patient’s documented history of severe allergic reaction to iodinated contrast media, contrast-enhanced CT was not performed. Non-contrast imaging revealed the largest lesion measuring approximately 27.0 × 12.6 cm, exhibiting heterogeneous density with prominent fatty components and scattered calcifications. This sizable mass exerted mass effect, displacing adjacent intestinal loops and the left lobe of the liver (**Figure 1**).

**Figure 2:**
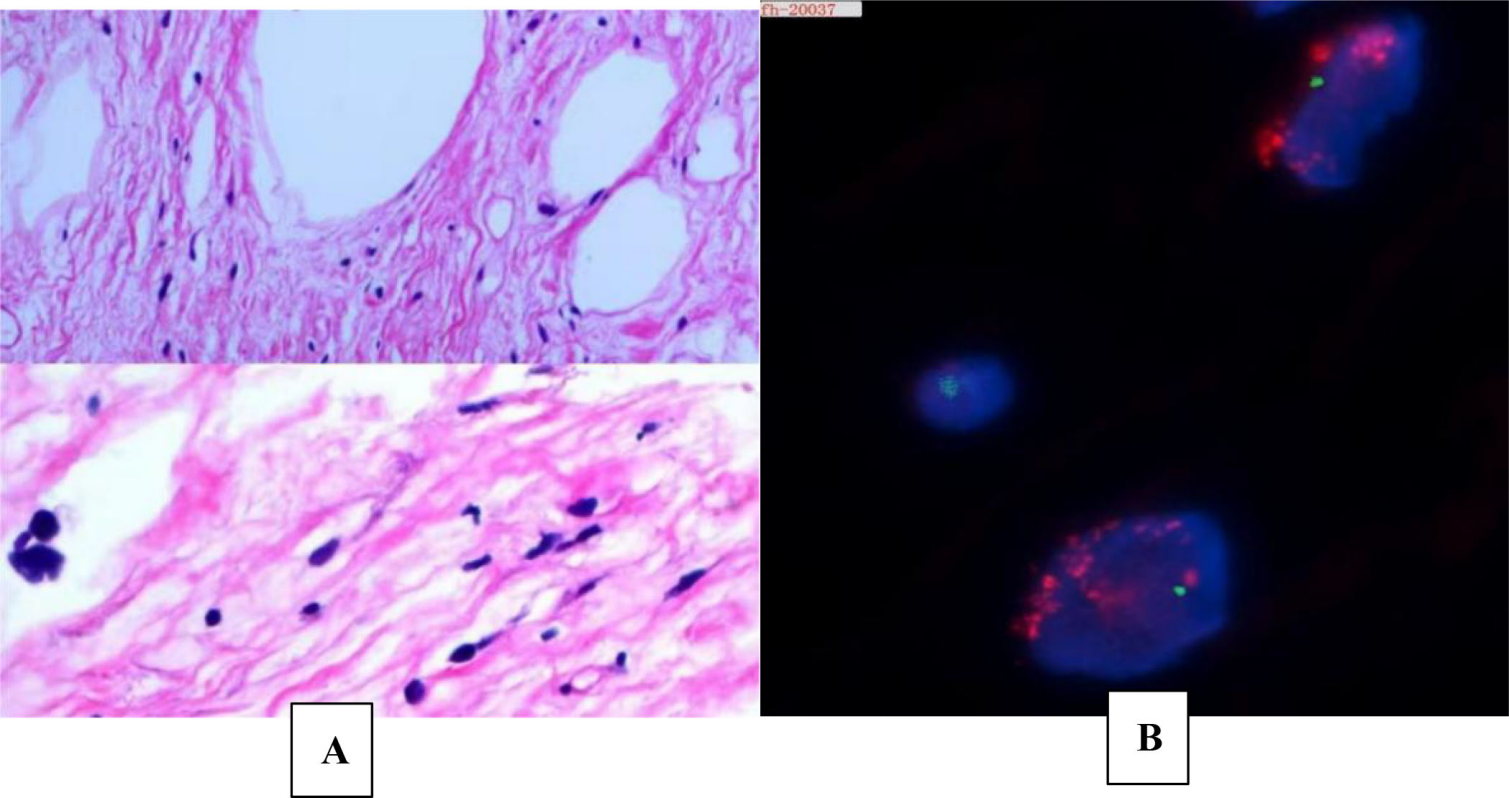
Pathology. Histopathological and fluorescence in situ hybridization (FISH) findings. Hematoxylin and eosin (H&E) staining **(A)**; FISH **(B)** [H&E, 100x].

In April 2020, an ultrasound-guided percutaneous biopsy of the retroperitoneal mass was performed. Pathological examination confirmed the diagnosis of WDLPS (**Figure 2A**). Subsequent fluorescence *in situ* hybridization (FISH) analysis confirmed amplification of the MDM2 gene. The average MDM2 copy number per cell was approximately 18.5, the CSP12 signal count was 1.1, and the MDM2/CSP12 ratio exceeded 2.0 (**Figure 2B**). Based on these comprehensive findings, the patient was diagnosed with retroperitoneal well-differentiated liposarcoma, staged as cT4N0M0 according to the American Joint Committee on Cancer (AJCC) staging system, 8th edition. It should be acknowledged that core needle biopsy, while diagnostically valuable, carries inherent sampling limitations due to tumor heterogeneity. Although the biopsy revealed pure WDLPS features, the presence of unsampled dedifferentiated components cannot be entirely excluded, particularly given the tumor’s large size and the absence of contrast-enhanced or functional imaging (e.g., 18F-FDG PET/CT) to guide biopsy targeting. This limitation is clinically relevant, as the presence of even focal dedifferentiation could alter prognosis and therapeutic response.

Notably, the patient presented with persistent and refractory hypoglycemia requiring long-term management with oral hypertonic glucose solutions. This was clinically suspected to represent a paraneoplastic phenomenon consistent with Doege-Potter syndrome, a condition most commonly associated with large mesenchymal tumors, including solitary fibrous tumors and, less frequently, sarcomas such as liposarcoma. Biochemical confirmation, including measurement of insulin-like growth factor-II (IGF-II) levels, was not performed due to the patient’s initial unstable condition and the limited availability of the assay at the time. Therefore, the diagnosis remains clinical rather than laboratory-confirmed. Nevertheless, the clinical context, including large tumor burden, fasting hypoglycemia without insulin therapy or pancreatic pathology, and rapid symptom improvement following glucose supplementation, supported this clinical suspicion. The presence of such a suspected paraneoplastic syndrome further complicated the clinical picture and underscored the metabolic impact of the tumor.

**Figure 3:**
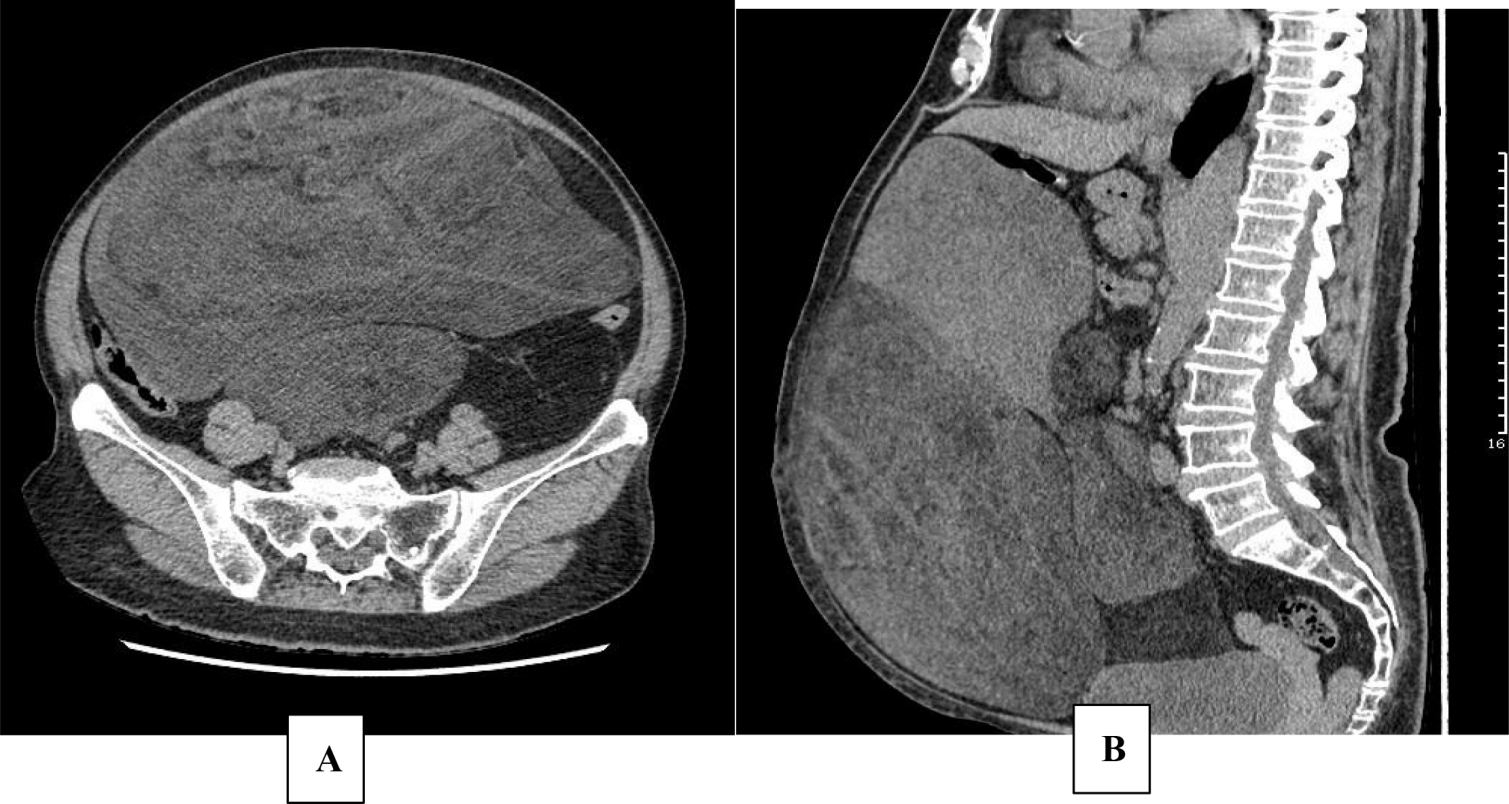
Abdominal CT. Abdominal CT obtained in August 2020 (after anlotinib therapy). Stable disease (SD) confirmed by imaging data. Axial **(A)**; Sagittal **(B)**.

A multidisciplinary team (MDT) specializing in soft tissue sarcoma reviewed the case. The patient underwent initial screening using the G8 tool, which yielded a score of 12 (below the cutoff of 14), indicating vulnerability and the need for further evaluation. Following this screening, a comprehensive geriatric assessment (CGA) was performed by the multidisciplinary team, including evaluations of functional status, comorbidity burden, polypharmacy, nutritional status, and cognitive function. The CGA confirmed the patient’s frailty profile and informed the treatment decision-making process, leading to the recommendation of a non-surgical approach with close monitoring for toxicity and selection of agents with favorable tolerability profiles. Consequently, systemic therapy was initiated. The patient commenced oral anti-angiogenic therapy with anlotinib at a dose of 12 mg once daily in the Department of Medical Oncology. During the treatment period from April to August 2020, the patient experienced a hypertensive crisis. Although blood pressure was eventually controlled with antihypertensive medication, it remained unstable, and proteinuria (2+) was noted. A dose reduction to 10 mg once daily was attempted to manage toxicity, but hypertension remained poorly controlled and proteinuria persisted. A follow-up CT scan in August 2020 was evaluated according to Response Evaluation Criteria in Solid Tumors (RECIST) version 1.1. The target lesion (the largest retroperitoneal mass) showed a 9.3% increase in the sum of the longest diameter compared to baseline (from 27.0 cm to 29.5 cm), which did not meet the RECIST 1.1 criteria for progressive disease (≥20% increase). Treatment efficacy was therefore formally assessed as stable disease (SD) (**Figures 3A, B**). However, given the slow but measurable tumor growth and the concurrent intolerable toxicity (hypertensive crisis, proteinuria), the decision was made to discontinue anlotinib.

**Figure 4:**
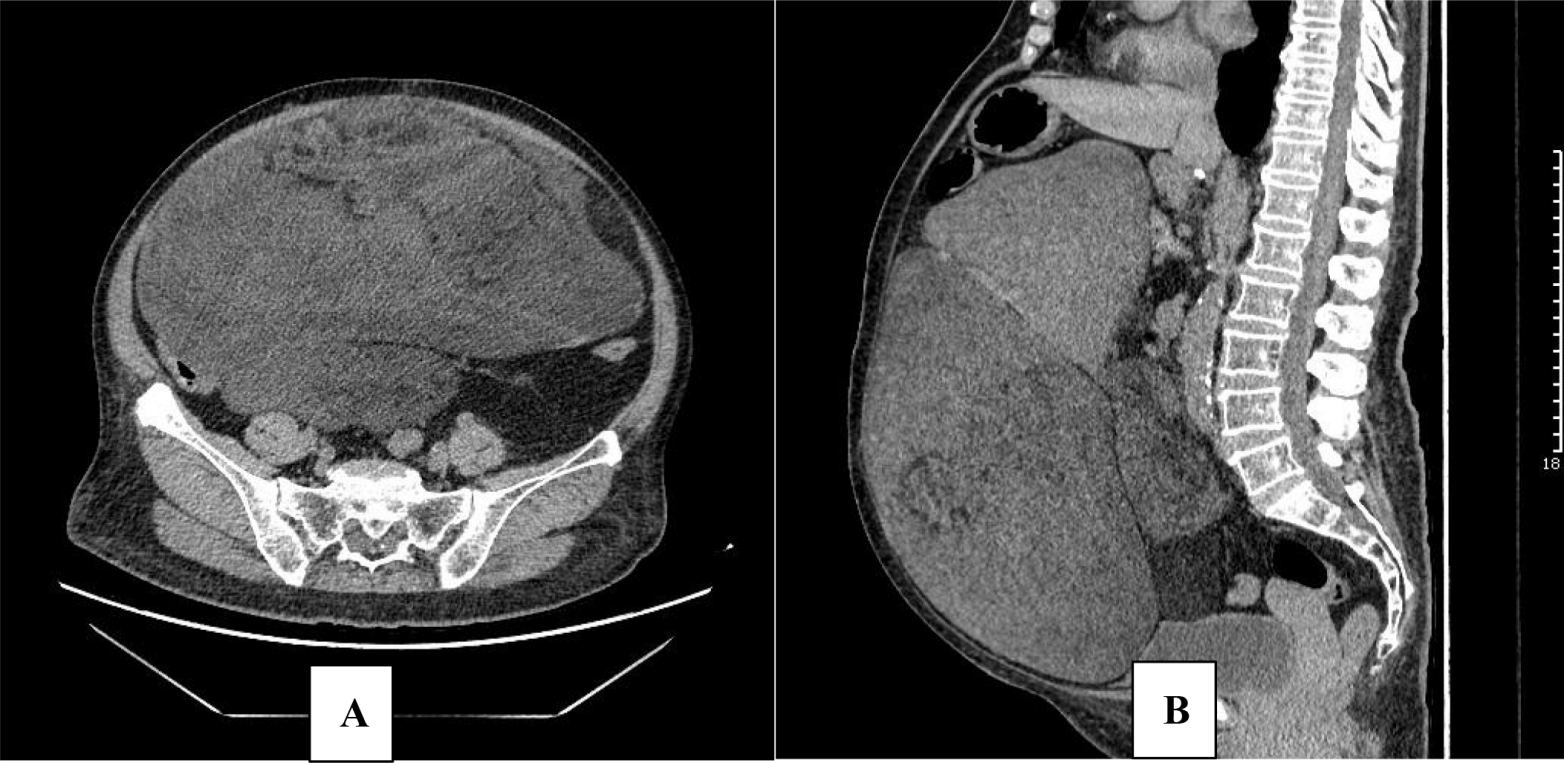
Abdominal CT. Abdominal CT obtained in October 2020 (cycle 2 of eribulin therapy). Stable disease (SD) confirmed by imaging data. Axial **(A)**; Sagittal **(B)**.

**Figure 5:**
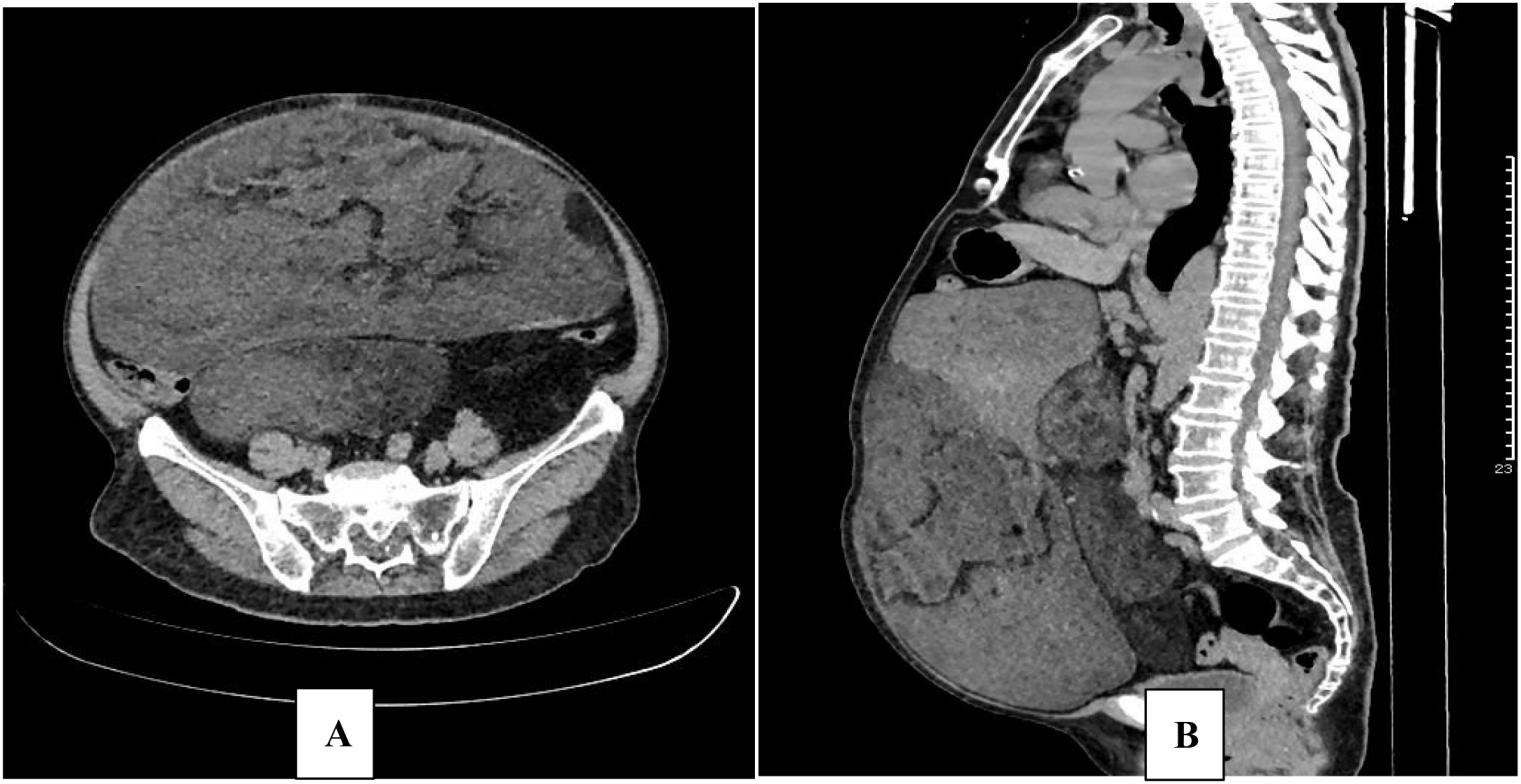
Abdominal CT. Abdominal CT obtained in July 2021 (cycle 16 of eribulin therapy). Stable disease (SD) confirmed by imaging data. Axial **(A)**; Sagittal **(B)**.

Following re-evaluation by the MDT, the treatment regimen was adjusted in September 2020. The patient was started on second-line therapy with eribulin at a dose of 1.4 mg/m², administered intravenously on days 1 and 8 of a 21-day cycle. Subsequent follow-up CT scans in October 2020 (**Figures 4A, B**) and July 2021 demonstrated continued stable disease (**Figures 5A, B**). Treatment has been ongoing to date. Throughout the five-year treatment period, the patient has remained on the full dose with no dose reductions required. Treatment has been well tolerated, with adverse events limited to Grade I–II leukopenia. No significant gastrointestinal reactions or neurotoxicity have been observed, and the patient’s blood pressure has been well-controlled.

To date, the patient has completed approximately 85 cycles of eribulin (21-day cycles over 5 years), with regular follow-up CT scans performed every 3–6 months. Throughout this extended treatment period, comprehensive safety monitoring has been conducted prior to each cycle, including complete blood counts, liver and kidney function tests, and clinical assessment for neurotoxicity. Adverse events have been consistently limited to Grade I–II leukopenia, with no evidence of cumulative toxicity, dose-limiting neurotoxicity, or treatment-related organ dysfunction. This favorable long-term safety profile has enabled uninterrupted full-dose administration and preserved the patient’s quality of life. Serial tumor measurements of the target lesion have demonstrated sustained stability throughout the treatment period, with variations remaining well below the 20% threshold for progressive disease (baseline: 27.0 cm × 12.6 cm; most recent: 27.2 cm × 12.9 cm). Importantly, there has been no evidence of new lesions, metastatic disease, or increase in solid non-lipogenic components. The patient’s disease has consistently been classified as stable disease (SD) according to RECIST 1.1 across all follow-up assessments. The patient maintains a good quality of life, with well-controlled blood glucose and blood pressure. A comprehensive timeline of the patient’s five-year treatment course is presented in **Table 1**.

**Table 1:** Timeline of key clinical events.

Date	Event
April 2020	Initial presentation with hypoglycemia and abdominal mass; CT imaging; biopsy-confirmed WDLPS with MDM2 amplification; initiation of anlotinib (12 mg daily)
August 2020	Hypertensive crisis and proteinuria; dose reduction to 10 mg daily; CT shows 9.3% increase in target lesion (SD per RECIST 1.1); anlotinib discontinued due to toxicity and slow progression
September 2020	Initiation of eribulin (1.4 mg/m² on days 1 and 8 of 21-day cycle)
October 2020	First follow-up CT after eribulin initiation; disease stability confirmed
July 2021	Follow-up CT; continued stable disease
Ongoing (March 2026)	Completed approximately 85 cycles of eribulin; sustained disease stability on serial CT imaging; maintained quality of life; no dose reductions required

## Follow-up and outcomes

Over five years of continuous eribulin therapy, the patient has maintained sustained disease stability with exceptional treatment adherence and tolerability. Serial CT imaging performed every 3–6 months consistently demonstrated stable disease per RECIST 1.1 criteria, with target lesion dimensions remaining nearly unchanged (baseline: 27.0 × 12.6 cm; most recent: 27.2 × 12.9 cm) and no evidence of new lesions, metastasis, or increased solid non-lipogenic components. The patient completed approximately 85 treatment cycles without dose reductions, experiencing only Grade I–II leukopenia and no cumulative toxicity, neurotoxicity, or gastrointestinal adverse effects. Clinically, she achieved well-controlled blood pressure and resolution of Doege-Potter syndrome-associated hypoglycemia, which had required long-term oral hypertonic glucose supplementation prior to tumor control. The patient reports excellent quality of life with minimal treatment-related discomfort and expresses high satisfaction with her disease management, underscoring the value of aligning therapeutic goals with patient-centered outcomes in elderly populations.

## Discussion

RLPS represents a relatively rare and clinically challenging subtype of soft tissue sarcoma, characterized by a complex anatomical location, high postoperative recurrence rates, and considerable therapeutic complexity. These challenges are particularly pronounced in elderly patients with multiple comorbidities, where treatment options are often limited and require careful balancing of efficacy and safety (13). In this report, we present the case of an 82-year-old female diagnosed with retroperitoneal WDLPS. Following a hypertensive crisis and evidence of slow tumor progression during first-line therapy with anlotinib, she was transitioned to second-line eribulin, and has since maintained prolonged disease stability exceeding five years. This case illustrates the potential for long-term disease control with eribulin in an elderly patient with inoperable WDLPS. While these findings are hypothesis-generating, they must be interpreted with particular caution given the exclusion of pure WDLPS from pivotal trials and the inherently indolent progression pattern of this subtype. A critical limitation of this case report is the inability to definitively disentangle the specific contribution of eribulin from the known indolent natural history of WDLPS, which can be associated with prolonged stable disease even in the absence of active systemic therapy. In the absence of a control group or a treatment-free observation period, the observed five-year disease stability should not be interpreted as direct evidence of a causal treatment effect, but rather as a clinical observation that suggests a possible therapeutic role for eribulin in this specific setting.

The diagnosis of RLPS requires a multimodal approach integrating imaging, histopathology, and molecular analysis, as illustrated in this case. Abdominal CT typically reveals a large, heterogeneous mass with predominant fat density, a characteristic radiological finding in RLPS (14). Beyond conventional imaging, volumetric 18F-FDG PET/CT provides valuable functional information that can guide surgical decision-making. Metrics such as metabolic tumor volume (MTV), total lesion glycolysis (TLG), and maximum standardized uptake value (SUVmax) have been shown to predict multifocality, histological grade, and pathological subtype preoperatively (15). However, imaging alone is insufficient to reliably differentiate WDLPS from benign lipomas or other retroperitoneal tumors. Thus, histopathological confirmation via ultrasound-guided percutaneous biopsy remains essential. In this case, pathological examination confirmed WDLPS, and the final classification was established through FISH, which identified MDM2 gene amplification, a highly specific molecular marker for WDLPS and DDLPS that is critical for distinguishing these entities from benign adipose tumors (16). While CDK4 amplification was not assessed in this case, it is important to note that FISH can reliably detect CDK4 amplification even in limited biopsy specimens, as it requires minimal tissue and is routinely performed alongside MDM2 testing. Assessment of CDK4 status could provide additional molecular profiling information and inform potential future use of CDK4/6 inhibitors, which represent a well-tolerated oral treatment option for advanced WDLPS/DDLPS. In retrospect, concurrent MDM2 and CDK4 FISH testing at diagnosis would have offered a more complete molecular characterization of this tumor (17–19). However, based on the strength of clinical evidence for eribulin and the need for prompt treatment switch following anlotinib toxicity, eribulin was selected as second-line therapy. Should disease progression occur, repeat biopsy for NGS testing to assess CDK4 amplification and other potential targets would be pursued to guide subsequent therapy, including the possible use of CDK4/6 inhibitors. Recently, emerging tools such as deep learning radiomics and CT-based radiomics models have demonstrated potential to non-invasively predict MDM2 status and tumor grade, offering promising avenues for more precise and less invasive diagnostic strategies in RLPS (20, 21). While the presence of calcifications on imaging may raise suspicion for a dedifferentiated component, histopathological examination of the biopsy specimen revealed pure WDLPS features with no evidence of non-lipogenic sarcomatous elements. Although sampling error due to tumor heterogeneity cannot be entirely excluded, the indolent clinical course, sustained disease control over five years on eribulin, and absence of aggressive radiological features argue against a significant DDLPS component. This distinction is clinically relevant, as response to systemic therapies may vary between subtypes. However, the durable response observed in this patient supports the diagnosis of WDLPS and underscores the potential efficacy of eribulin in well-differentiated disease.

The prognostic and therapeutic implications of histological subtyping in RLPS cannot be overstated. WDLPS and DDLPS, despite sharing MDM2 amplification, represent distinct biological entities with different clinical trajectories and treatment responses. The well-differentiated histology in this patient likely provided a biological basis for her sustained response to eribulin, as WDLPS typically exhibits indolent growth and may be more susceptible to cytostatic agents that disrupt microtubule dynamics (22). Another crucial prognostic variable is the response to systemic treatment. In patients with high-risk soft tissue sarcoma receiving neoadjuvant chemotherapy, treatment response has been clearly correlated with improved disease-free survival (DFS) and OS (23). Additionally, systemic inflammatory indices have been identified as independent predictors of RFS and locoregional recurrence-free survival (LRFS) in RLPS patients (24). Recent studies have further elucidated the molecular underpinnings of this differential response. Grimaudo et al. (12) prospectively evaluated eribulin in advanced liposarcoma and demonstrated that treatment response correlated with alterations in tumor vascularity and metabolic activity on functional imaging, with WDLPS patients showing more durable disease control. In contrast, DDLPS, particularly when harboring additional molecular alterations such as CDK4 amplification or mutations in tumor suppressor genes, may exhibit more variable responses to eribulin. This heterogeneity underscores the importance of molecular profiling to guide treatment selection. While CDK4 amplification was not assessed in this case due to limited biopsy material, future studies should prioritize comprehensive genomic profiling to identify biomarkers predictive of eribulin sensitivity in both WDLPS and DDLPS. Recent advances in computational approaches have enabled drug sensitivity prediction by integrating genomic and chemical features, with emerging machine learning models showing promise in sarcoma cell lines (25, 26). Although these data-driven frameworks hold potential for guiding treatment decisions, their applicability to rare and heterogeneous malignancies such as retroperitoneal liposarcoma remains underexplored and warrants further investigation. The prolonged stable disease achieved in this patient, sustained for over five years on eribulin, is exceptional and raises the intriguing possibility that a subset of WDLPS patients may harbor molecular features conferring heightened sensitivity to this agent. Emerging research into the tumor microenvironment, microtubule-binding protein expression, and IGF-2 signaling may provide further insights into the mechanisms underlying durable responses.

Surgical resection with negative margins (R0) remains the only potentially curative approach for RLPS (27). However, as exemplified in this case, factors such as advanced age, large tumor size, anatomical proximity to critical structures, and associated paraneoplastic conditions (refractory hypoglycemia) may preclude curative surgery. In such scenarios, a MDT evaluation is essential to determine unresectability and guide appropriate systemic therapy. For unresectable or advanced RLPS, systemic treatment options include chemotherapy (e.g., doxorubicin), targeted therapy (e.g., anlotinib), and microtubule inhibitors such as eribulin (28). Notably, immune checkpoint inhibitors have shown promising activity in other sarcoma subtypes, such as DDLPS and undifferentiated pleomorphic sarcoma (UPS), particularly when combined with radiotherapy in the neoadjuvant setting (29). However, their role in RLPS remains less defined and requires further study.

In this patient, first-line therapy with anlotinib was complicated by adverse events, such as hypertensive crisis and proteinuria, and exhibited limited efficacy with slow radiological progression. Despite a dose reduction to 10 mg daily, toxicity persisted and tumor growth continued, prompting a timely switch to second-line eribulin, which resulted in sustained disease control. Eribulin, a microtubule dynamics inhibitor, has established efficacy in liposarcoma, as supported by prior clinical studies. A study in advanced liposarcoma patients receiving eribulin monotherapy documented a PFS of 3.3 months, OS of 8.7 months, and with manageable toxicities including peripheral neuropathy and neutropenia (12). Another clinical study evaluated the combination of eribulin and pembrolizumab in patients with metastatic soft tissue sarcoma and demonstrated manageable toxicity, with 12-month PFS rates of 36.8% in the leiomyosarcoma cohort and 69.6% in the liposarcoma cohort (30). The present case extends these findings by demonstrating the feasibility of long-term administration and durable disease control in an elderly patient, reinforcing the clinical utility of eribulin in this population.

An important aspect of this case is the presence of refractory hypoglycemia as a suspected paraneoplastic manifestation clinically consistent with Doege-Potter syndrome. This syndrome is most frequently reported in association with solitary fibrous tumors, but it has also been described in other mesenchymal neoplasms, including sarcomas (31). The underlying mechanism involves tumor secretion of high molecular weight insulin-like growth factor-II (IGF-II), which aberrantly activates insulin receptors, leading to hypoglycemia (32). In this case, biochemical confirmation, such as elevated IGF-II levels, was not obtained due to the patient’s acute presentation and assay limitations. Accordingly, the diagnosis remains clinical rather than laboratory-confirmed. Nevertheless, the clinical features, including large tumor burden, fasting hypoglycemia with resolution upon glucose administration, and absence of other identifiable causes, strongly supported this clinical suspicion. The recognition of Doege-Potter syndrome in sarcoma patients is clinically important, as it may necessitate additional supportive measures and can sometimes resolve with effective tumor debulking or systemic therapy (33). Interestingly, preclinical evidence in other tumor types has suggested a potential interaction between IGF signaling and eribulin sensitivity (34). Whether such mechanisms are relevant in liposarcoma, or in the context of Doege-Potter syndrome, remains entirely speculative and warrants further investigation. Alternatively, it is possible that effective tumor control with eribulin contributed to metabolic stabilization, a hypothesis supported by reduced IGF-II production, though this was not directly measured in our patient. Further research is needed to explore the relationship between IGF signaling and response to eribulin in liposarcoma. In this patient, the hypoglycemia, which was managed with long-term oral hypertonic glucose solutions, persisted until disease stabilization was achieved with eribulin. This further highlights the tumor’s metabolic activity and the systemic impact of disease control.

Treatment of elderly sarcoma patients necessitates careful consideration of both efficacy and tolerability. Advanced age is an established adverse prognostic factor in liposarcoma (35), and conventional first-line anthracycline-based chemotherapy is often poorly tolerated in older adults due to cardiotoxicity and myelosuppression (36, 37). Comprehensive geriatric and functional assessment is therefore essential to tailor therapy to individual capacity and comorbidities. Japanese studies also demonstrated that for advanced soft tissue sarcomas, patient age, gender, histological subtype, tumor location, treatment, disease extent, and 3-year OS are significantly correlated (38). Alternative agents such as trabectedin and pazopanib have been evaluated in older patients, with the EPAZ study demonstrating non-inferiority of pazopanib compared to doxorubicin in patients aged >60 years (39, 40). The exceptional duration of response observed in this patient, exceeding five years, differs notably from the median progression-free survival of 2.9 months reported for liposarcoma patients in the phase III eribulin trial (11). Several factors may explain this discrepancy: (1) Histological subtype: Pure WDLPS typically exhibits indolent growth and may be more susceptible to cytostatic agents like eribulin than mixed or dedifferentiated subtypes. (2) Tumor kinetics: The slow doubling time of WDLPS means that even modest cytostatic effects can result in prolonged radiographic stability. (3) Treatment adherence: The patient received uninterrupted full-dose eribulin for five years, maximizing cumulative drug exposure. (4) Tumor biology: The presence of Doege-Potter syndrome suggests active IGF-2 signaling, which may intersect with eribulin’s mechanism in ways not yet fully understood. (5) Trial population differences: Clinical trial patients often have more aggressive disease and higher tumor burden than real-world long-term responders. Understanding such exceptional responses may inform future biomarker-driven patient selection for eribulin therapy. Furthermore, the individualized approach adopted in this case exemplifies several key principles in the management of elderly RLPS: (1) initial selection of an oral agent (anlotinib) to minimize treatment burden and toxicity. (2) prompt adaptation upon development of intolerable adverse events. (3) transition to a drug with established efficacy and a manageable safety profile (eribulin), where toxicity—primarily myelosuppression—was easily monitored and controlled. (4) alignment of treatment goals with a “chronic disease” model aimed at long-term tumor control and preservation of quality of life rather than curative intent.

Importantly, the patient’s own perspective, characterized by sustained quality of life, minimal treatment-related discomfort, and satisfaction with disease stability, underscores the value of aligning therapeutic goals with patient-centered outcomes in elderly populations.

## Conclusion

Molecular profiling plays an important role in guiding treatment decisions in RLPS. For select patients with advanced WDLPS, eribulin may represent a well-tolerated option for long-term disease control, particularly when surgery is not feasible. However, given the exploratory nature of this observation and the exclusion of pure WDLPS from pivotal trials, further research is needed to confirm its efficacy in this subtype. The MDT framework and individualized treatment planning remain essential in navigating the complexities of such cases, requiring ongoing evaluation of therapeutic response and toxicity to guide timely modifications. In this patient, stabilization of paraneoplastic hypoglycemia (Doege-Potter syndrome) coincided with disease control achieved during eribulin therapy, suggesting potential systemic metabolic benefits of tumor control. This case demonstrates the possibility of prolonged disease stability during eribulin treatment in an elderly patient with inoperable retroperitoneal WDLPS. Nevertheless, given the inherent limitations of a single case report, including potential sampling bias from core needle biopsy, the indolent natural history of WDLPS, and the absence of a control group or treatment-free observation, these findings should be considered hypothesis-generating rather than confirmatory of a causal treatment effect. The observations underscore the importance of multidisciplinary assessment, comprehensive geriatric evaluation, and individualized treatment planning in managing this challenging patient population. Further research is needed to validate the efficacy of eribulin in pure WDLPS and to identify molecular predictors of response.

## Data Availability

The datasets presented in this study can be found in online repositories. The names of the repository/repositories and accession number(s) can be found in the article/Supplementary Material.
